# HDAC1-3 inhibition triggers NEDD4-mediated CCR2 downregulation and attenuates immunosuppression in myeloid-derived suppressor cells

**DOI:** 10.1007/s00262-024-03931-y

**Published:** 2025-02-01

**Authors:** Zhiqi Xie, Jinjin Shao, Zeren Shen, Zhichao Ye, Yoshiaki Okada, Daisuke Okuzaki, Naoki Okada, Masashi Tachibana

**Affiliations:** 1 Wuyi First People’s Hospital, Affiliated Hospital, School of Medicine, Hangzhou City University, Hangzhou, 310015 China; 2 Project for Vaccine and Immune Regulation, Graduate School of Pharmaceutical Sciences, Osaka University, Osaka, 565-0871 Japan; 3 Key Laboratory of Drug Safety Evaluation and Research of Zhejiang Province, Center of Safety Evaluation and Research, Hangzhou Medical College, Hangzhou, 310053 China; 4 Department of Plastic Surgery, The First Affiliated Hospital, School of Medicine, Zhejiang University, Hangzhou, 310003 China; 5 Laboratory of Clinical Science and Biomedicine, Graduate School of Pharmaceutical Sciences, Osaka University, Osaka, 565-0871 Japan; 6 Laboratory of Human Immunology (Single Cell Genomics), WPI Immunology Frontier Research Center, Osaka University, Osaka, 565-0871 Japan; 7 Laboratory for Context-Dependent Cell Immunology, Department of Biomedical Sciences, College of Life Sciences, Ritsumeikan University, 1-1-1 Nojihigashi, Kusatsu, Shiga 525-8577 Japan

**Keywords:** Myeloid-derived suppressor cells, Histone deacetylase, NEDD4, CCR2, Hepatocellular carcinoma

## Abstract

Myeloid-derived suppressor cells (MDSCs) play a critical role in cancer progression and resistance, thus representing promising targets for immunotherapy. Despite the established role of histone deacetylases (HDACs) in epigenetic regulation of cell fate and function, their specific impact on MDSCs remains elusive. We sought to investigate the effects and underlying mechanisms of HDAC on MDSCs using various HDAC inhibitors. Our results indicate that HDAC1-3 inhibitors reduce CCR2 expression, a chemokine receptor that mediates the migration of monocytic (M-)MDSCs to tumors and attenuated the immunosuppressive activity of MDSCs. In an orthotropic hepatocellular carcinoma (HCC) murine model, HDAC1-3 inhibitors reduced the infiltration of M-MDSCs, increased the number of natural killer cells in tumors, and suppressed tumor growth. Our results also suggest that HDAC1-3 inhibitors potentiate the antitumor effects of anti-programmed cell death protein 1 antibodies. ATAC-seq and RNA-seq analyses revealed 115 genes epigenetically upregulated by HDAC1-3 inhibitors, primarily linked to transcriptional regulation and ubiquitination. We further elucidated that HDAC1-3 inhibitors facilitate CCR2 protein degradation through ubiquitination-mediated by NEDD4 E3 ligase. Our findings reveal a novel mechanism of action of HDAC1-3 inhibitors in MDSCs and suggest a potential synergistic immunotherapy strategy for clinical benefit in HCC.

**Supplementary Information:**

The online version contains supplementary material available at 10.1007/s00262-024-03931-y.

## Introduction

The dysfunction or exhaustion of antitumor immune cells, such as effector T and natural killer (NK) cells, can facilitate immune evasion and tumor progression [1]. Immunotherapy is a promising approach for cancer treatment, particularly immune checkpoint blockade (ICB) therapy, which can reinvigorate antitumor immunity by alleviating the immunosuppression of tumor cells against antitumor immune cells [2–4]. However, most patients still show limited or no response to ICB therapy, probably because it is less effective in relieving immunosuppression mediated by other cells, such as myeloid-derived suppressor cells (MDSCs) [5–7]. MDSCs are a heterogeneous population of myeloid progenitor and immature myeloid cells that are partially blocked in their differentiation and development under tumor conditions, forming two phenotypes: monocytic (M-)MDSCs and polymorphonuclear (PMN-)MDSCs [8]. They inhibit antitumor immune responses by secreting regulatory factors, altering immune metabolism, and expressing immune checkpoints, which play crucial roles in the pathogenesis, recurrence, metastasis, immune escape, and drug resistance of multiple cancers [6, 9–11]. Studies have revealed that MDSC levels increase in patients with cancer who are resistant to ICB therapy, suggesting that MDSCs promote tumor immune escape via a mechanism distinct from ICB therapy, and that targeting MDSCs could improve the response rate to immunotherapy [12, 13].

Epigenetic regulation plays crucial roles in cancer progression and immune cell function. Histone deacetylases (HDACs) are key molecules that regulate cell fate by removing acetyl groups from lysine residues at the N-termini of histones, modulating gene transcription and expression. Several studies have reported the effects of HDACs on MDSCs. HDACs in mammals are classified into four classes: Zn^2+^-dependent class I (HDAC1, 2, 3, and 8), class IIa (HDAC4, 5, 7, and 9), class IIb (HDAC6 and 10), class IV (HDAC11) HDACs, and NAD^+^-dependent class III (Sirt1-7) HDACs, with distinct structural features [14]. HDAC11 deficiency enhances the immunosuppressive activity of MDSCs in tumor-bearing mice [15]. Hashimoto et al*.* reported that MS-275, an inhibitor of HDAC1-3, reduced the immunosuppressive activity of PMN-MDSCs, whereas ricolinostat, an inhibitor of HDAC6, reduced the immunosuppressive activity of M-MDSCs in tumor-bearing mice [16]. Our previous study revealed that valproic acid (VPA), a class I/IIa HDAC inhibitor (HDACi), significantly reduced the accumulation of M-MDSCs in tumors and attenuated PMN-MDSC immunosuppression [10]. Thus, different HDACs play distinct roles in the regulation of MDSCs. However, the effects and mechanisms underlying MDSC regulation by HDAC remain largely unknown. This study aimed to identify the types of HDACs that are important for regulating the accumulation and immunosuppression of MDSCs in tumors.

Hepatocellular carcinoma (HCC) accounts for 75%–85% of liver cancers, with over 80% of patients diagnosed at an advanced stage, and a 5-year survival rate of only 10%–18% [17]. The first-line systemic treatment for advanced HCC shifted from multikinase inhibitors, such as sorafenib, to immunotherapy after the Food and Drug Administration approved the combination of a programmed death-ligand 1 monoclonal antibody (atezolizumab) and a VEGF antagonist (bevacizumab) in 2020. However, the objective response rate of immunotherapy is only approximately 30%, which is significantly higher than that of sorafenib (13%); however, 70% of patients with HCC remain unresponsive [18]. Therefore, the role and molecular mechanisms of tumor microenvironments in HCC immune evasion needs to be explored. MDSCs contribute to immune evasion and resistance in HCC. For example, MDSC elimination in patients with hepatitis can prevent or delay HCC development [19]. Tumor-infiltrating MDSCs inhibit effector T cells, mediate HCC immune escape, and promote tumor recurrence and metastasis [20, 21]. MDSC levels were found to increase in patients with HCC resistant to ICB therapy; thus, targeting MDSCs could improve immunotherapy outcomes [22, 23]. Therefore, we explored whether strategies targeting HDACs in MDSCs could enhance the efficacy of existing HCC therapies.

## Materials and methods

### Cell lines

The Hepa 1–6 (RCB1638) cell line was obtained from the RIKEN CELL BANK. The cells were thawed and cultured according to the RIKEN CELL BANK guidelines. Dulbecco’s modified Eagle medium (FUJIFILM Wako, Japan) containing 10% fetal bovine serum (FBS) (Gibco, USA) and 1% antibiotic–antimycotic mixed stock solution (100 ×) (Nacalai Tesque, Japan) was used. The cells were cultured in a CO_2_ incubator at 37 °C, saturated vapor pressure, and 5% CO_2_ and used within 1 month of thawing from the early passage (less than three passages in the original vial).

### Experimental animals

The C57BL/6 J mice were purchased from Japan SLC (Shizuoka, Japan). They were bred in a specific pathogen-free environment, and 6–8-week-old female mice were selected in the experiments. All animal experiments were conducted in compliance with regulations after obtaining approval from the Osaka University Experimental Animal Committee.

### In vitro MDSC differentiation

The bone marrow (BM) cells were differentiated into MDSCs in vitro. Briefly, BM cells from C57BL/6 J mice were stimulated with 40 ng/mL recombinant GM-CSF (Peprotech, NJ, USA) for 4 days. MS-275 (Carbosynth, UK), TC-H 106 (Tocris, UK), bufexamac (FUJIFILM, Japan), CXD101, TMP269, PCI-34051, Panobinostat, SAHA, TSA (MedChemExpress, USA), and SIS17 (Selleck, USA) were dissolved in dimethyl sulfoxide (DMSO) (10 mM), diluted with RPMI 1640 medium, and added at the initiation of in vitro MDSC differentiation. In examining the proteolytic system, MS-275 or CXD101 was added (250 nM) on day 4 of in vitro MDSC differentiation, MG-132 (5 µM; Selleck), 3-methyladenine (MA) (1 mM; Selleck) and cycloheximide (CHX) (10 µg/mL; MedChemExpress) were added at the same time as the above HDAC inhibitor. In vitro MDSCs were harvested at the indicated time points and subjected to flow cytometry.

### Flow cytometry

After washing various cells with 2% FBS/phosphate buffered saline (PBS), mononuclear cells were treated with TruStain FcX (anti-mouse CD16/32) antibody (Clone 93, BioLegend, USA) and incubated at 4 °C for 5 min to block Fc receptors. The following fluorescence-labeled antibodies were then allowed to react at 4 °C under light-shielding conditions for 15 min. The cells were then washed twice with 2% FBS/PBS. The 7-amino-actinomycin D Viability Staining Solution (BioLegend) was added to eliminate dead cells, and we analyzed surface antigen expression using a flow cytometer (BD FACSCanto II, BD Biosciences, USA). Data were analyzed using the FlowJo software (BD Biosciences). The following antibodies were used: allophycocyanin (APC)-labeled anti-mouse CD11b (Clone M1/70, eBioscience, USA), Pacific Blue-labeled anti-mouse Gr-1 (Clone RB6-8C5), fluorescein isothiocyanate (FITC)-labeled anti-mouse Ly-6G (Clone 1A8), APC-Cy7-labeled anti-mouse Ly-6C (Clone HK1.4), Pacific Blue-labeled anti-mouse CD45 (Clone 30-F11), FITC-labeled anti-mouse CD8α (Clone 53–6.7), FITC-labeled anti-mouse CD3ε (Clone 145-2C11), APC-labeled anti-mouse NK1.1 (Clone PK136), and phycoerythrin (PE)-labeled anti-mouse CCR2 (Clone SA203G11).

### Analysis of immunosuppressive function of MDSCs

CD4^+^ and CD8^+^ T cells were isolated from the spleen lymphocytes of healthy mice using MojoSort mouse CD4 nanobeads and a MojoSort mouse CD8α selection kit (BioLegend), according to the manufacturer’s protocol. The cells were suspended in RPMI 1640 medium (10% FBS, 2 mM GlutaMAX, 100 units/mL penicillin–streptomycin, 10 mM 4-(2-hydroxyethyl)-1-piperazineethanesulfonic acid, 100 μM nonessential amino acids, 1 mM sodium pyruvate, 55 μM 2-mercaptoethanol; Gibco) and seeded at 1 × 10^5^ cells/well in a 96-well plate. T cell proliferation was stimulated with anti-CD3ε (1 μg/mL)/CD28 (1 μg/mL) antibodies (BioLegend). The anti-CD3ε antibody was coated on each well of the plate via overnight incubation at 4℃ with PBS before coculture. CD4^+^ and CD8^+^ T cells were labeled with eFlour670 (eBioscience) and cocultured with in vitro MDSCs. The cell proliferation rate was analyzed after three days using flow cytometry.

### Establishment of orthotropic HCC mouse model

Hepa 1–6 cancer cells were transplanted into the liver of C57BL/6 J mice as previously described [24]. Mice were shaved and anesthetized with isoflurane using an experimental animal anesthesia device. They were placed in a supine position on a heat plate, and their abdomens were opened using a median incision. The middle lobe of the liver was exposed to a sterile cotton swab, and 20 µL of Hepa 1–6 cells/matrigel solution (Corning) (5 × 10^7^ cells/mL) was injected using a microsyringe (100 µL; Agilent, Japan). The injection site was pressed with a sterile cotton swab to stop bleeding and the middle lobe was returned to its original position. Finally, the abdomen was closed by continuous suturing of the abdominal wall with curved suture needles (Natsume Seisakusho), and the epidermis was fixed with an experimental animal automatic suture device (using forceps and suture clips of 9 mm, Natsume Seisakusho). Ten days after the inoculation of Hepa 1–6 cells, mice with a MDSC proportion in the blood greater than 20% were selected for further experimentation (Fig. S3A). The vehicle solution or MS-275 (5 mg/kg; Selleck) and CXD101 (5 mg/kg; MedChemExpress) were intraperitoneally administered daily for 10 days. MS-275 and CXD101 were prepared by dissolving them in 1% DMSO, 15% PEG300, and PBS. Anti-PD-1 antibody (Clone: J43) or IgG antibody (200 µg/mouse, Bio X Cell) was intraperitoneally administered every 3 days from 10 days after the inoculation of Hepa 1–6 cells. On day 21 after Hepa 1–6 transplantation, the livers were removed and photographed. The hard, white tumors were then separated from the livers, and their weights were measured.

### Mononuclear cell isolation

Spleen: mice spleens were collected and the cells were dispersed on a 70-µm cell strainer. After centrifugation at 330 × g, 4℃ for 5 min, the supernatant was aspirated. The obtained cells were resuspended in ammonium–chloride–potassium (ACK) lysis buffer to remove red blood cells and washed with 2% FBS/PBS to obtain splenic mononuclear cells.

Tumor: mice tumors were finely minced using scissors and dispersed in 100 units/mL collagenase I (Funakoshi, Japan), 2% FBS/PBS, and incubated at 37 °C for 30 min for cell dispersion. The tissue fragments were removed using a 70-µm cell strainer and after centrifugation at 330 × g, 4 °C for 6 min, the supernatant was aspirated. The cells were suspended in ACK lysis buffer to remove red blood cells and washed with 2% FBS/PBS to obtain mononuclear tumor cells.

Peripheral blood: peripheral blood collected from mice fundi was suspended in ACK lysis buffer to remove red blood cells and after centrifugation at 400 × g, 4 °C for 5 min, the supernatant was aspirated. This procedure was repeated once to obtain peripheral blood mononuclear cells.

### ATAC-seq analyses

M-MDSCs (CD11b^+^Ly-6C^hi^Ly-6G^−^; purity > 95%) were sorted from isolated tumors using JSAN (KS-Techno, Japan). ATAC-seq libraries were prepared using an ATAC-seq Kit (Active Motif, USA), according to the manufacturer’s protocol. Briefly, 1 × 10^5^ M-MDSCs were lysed with ATAC lysis buffer and nuclei were centrifuged at 500 × g, 4 °C for 10 min. Nuclei were then suspended in 50 µL of Tagmentation Master Mix and incubated at 37 °C for 30 min with shaking. DNA was purified using a DNA purification column and polymerase chain reaction (PCR) was performed for 10 cycles to introduce sequencing adapters into the open chromatin regions. The library DNA was purified using solid-phase reversible immobilization beads. Molecular weights were checked, and libraries were quantified using a Bioanalyzer and submitted for next-generation sequencing (NGS) to the Genome Information Research Center, Research Institute for Microbial Diseases, Osaka University. FASTQ files were received and ATAC-seq data were analyzed using Active Motif’s Basepair Portal web tool with an automated pipeline. The ATAC-seq libraries had an average of > 70 million reads per sample. Sequenced reads were mapped to the mouse reference genome mm10 using Bowtie2, and differential analyses and gene annotation were performed using MACS2 (default options: no model; *p* < 0.00001; shift: 0). In this analysis, promoters were defined as those located 5 kb upstream of the transcription start site. ATAC-seq tracks were visualized using the Integrative Genomics Viewer (v.2.11.0).

### RNA-seq analyses

Total RNA was extracted from M-MDSCs sorted from tumors using the miRNeasy Mini Kit (Qiagen, Germany). RNA libraries were prepared using TruSeq stranded mRNA sample prep kit (Illumina, USA) and sequenced on Illumina NovaSeq 6000 platform (101 bp single-end mode). RNA extraction for NGS was outsourced to the Genome Information Research Center of the Research Institute for Microbial Diseases, Osaka University, Japan. RNA-seq data were analyzed using the BioJupies web tool and a volcano plot was generated. Genes with adjusted *p* < 0.05 and fold change > 1.5 were extracted as significantly differentially expressed genes.

### Quantitative real-time (qRT)-PCR

Total RNA was extracted from M-MDSCs sorted from tumors or in vitro MDSCs using TRIzol reagent (Thermo Fisher Scientific, USA), and reverse transcription was performed using the QuantiTect Reverse Transcription Kit (QIAGEN, Germany). The cDNA of each gene was amplified using TB Green Premix Ex Taq II (TaKaRa, Japan), and PCR was performed using the CFX96 Real-Time PCR system (Bio-Rad, USA). Expressions were compared using the ∆∆Ct method and mRNA expressions were normalized to those of mouse *Gapdh* mRNA. The primers used are listed as follows: *Gapdh*, 5ʹ-TGACCTCAACTACATGGTCTACA-3ʹ (forward); 5ʹ-CCGTGAGTGGAGTCATACTGG-3ʹ (reverse). *Ccr2*, 5ʹ-ATCCACGGCATACTATCAACATC-3ʹ (forward); 5ʹ-CAAGGCTCACCATCATCGTAG-3ʹ (reverse). *Nedd4*, 5ʹ-TCGGAGGACGAGGTATGGG-3ʹ (forward); 5ʹ-GGTACGGATCAGCAGTGAACA-3ʹ (reverse).

### Western blot

In vitro MDSCs were harvested and lysed in RIPA buffer (Beyotime, China) containing protease and phosphatase inhibitors for 30 min on ice. The lysates were then centrifuged at 14,000 rpm for 15 min at 4 °C, and the supernatants were collected and determined protein concentrations using the BCA protein assay (Thermo Scientific). Equal amounts of each sample diluted in 5 × SDS loading buffer were subjected to SDS polyacrylamide gel electrophoresis and then transferred to polyvinylidene fluoride (PVDF) membranes (Millipore). The membranes were blocked with 5% nonfat dry milk in TBST for 2 h, followed by overnight incubation with primary antibodies at 4 °C. The primary antibodies used were *β*-actin (1:20,000, cat no. 66009, Proteintech, China), NEDD4 (1:1000, cat no. 21698, Proteintech), CCR2a (1:1000, cat no. 16153, Proteintech), and ubiquitin (1:1000, cat no. 10201, Proteintech). After three washes with TBST, the membranes were incubated with HRP-labeled secondary antibodies (Beyotime). The protein levels were detected using an ECL-plus kit (Beyotime) and visualized using autoradiography film (Clinx, China).

### Immunoprecipitation and immunoblotting

Immunoprecipitation was conducted using the Immunoprecipitation Kit with Protein G Magnetic Beads (Beyotime). Briefly, cells were washed with cold PBS and lysed in lysis buffer supplemented with a protease inhibitor cocktail. Protein-G Magnetic beads were incubated with the primary antibodies for NEDD4 (50 μg/mL, cat no. 21698, Proteintech) or CCR2 (50 μg/mL, cat no. 16153, Proteintech), respectively, and rotated gently for 1 h at room temperature. Subsequently, the cell lysate was mixed with the antibody-bound Protein-G Magnetic beads and gently rotated overnight at 4 °C. The immunoprecipitates were then washed three times with wash buffer to remove unbound proteins. Proteins were recovered by boiling the beads in 5 × SDS loading buffer and analyzed by western blotting as described previously.

### Immunofluorescence staining

After being treated with CXD101 (250 nM) and MG-132 (5 µM) for 6 h, in vitro MDSCs were harvested and washed with ice-cold PBS three times, then fixed in 4% paraformaldehyde for 30 min. To permeabilize the cells, 0.1% Triton X-100 was added for 15 min at room temperature, and 3% BSA was used to block cells for 30 min. Cells were stained with a primary antibody against NEDD4 (1:200, cat no. 21698, Proteintech) and CCR2 (1:2000, cat no. 16153, Proteintech). CCR2 staining is pseudo-colored red (Cy3), and NEDD4 staining is pseudo-colored green (Alexa Fluor 488). The nuclei were stained with 4’,6-diamidino-2-phenylindole (DAPI). Immunofluorescent images were recorded using a fluorescence scanning device (3DHISTECH, Sysmex, Switzerland).

### Lentivirus construction and cell transduction

Lentiviral constructs for *Nedd4*-specific shRNA (sh-Nedd4) and negative control shRNA (sh-NC) were prepared by Shanghai GeneChem (Shanghai, China), with sequences 5′-GCTCAAGAAGCAGACTGACAT-3′ and 5′-TTCTCCGAACGTGTCACGT-3′, respectively, and titers of 1 × 10^9^ TU/mL. BM cells (1 × 10^5^ per well) were seeded in 48-well plates and transduced with lentiviruses at a multiplicity of infection (MOI) of 400, supplemented with HitransG A and GM-CSF (40 ng/mL). After 72 h of incubation at 37°C in a 5% CO_2_ incubator, the efficiency of shRNA was determined by GFP expression and qRT-PCR for *Nedd4* knockdown, followed by treatment with CHX (10 µg/mL) and CXD101 (250 nM) for 12 h before flow cytometry analysis.

### Statistical analyses

GraphPad Prism (GraphPad Software, USA) was used for statistical analyses and *p*-values were calculated using Student’s *t* test, one-way ANOVA, two-way ANOVA, log-rank test, or Pearson’s correlation coefficient test.

### Data availability statement

NGS data are available in GEO under the accession number GSE232268. All data are available from the corresponding authors upon request.

### Ethics approval

This study protocol was reviewed and approved by [The Animal Experiment Committee of Osaka University], approval number [Douyaku R03-7–2].

## Results

### HDAC1-3 inhibitors reduce CCR2 expression and the immunosuppressive function of MDSCs

**Fig. 1:**
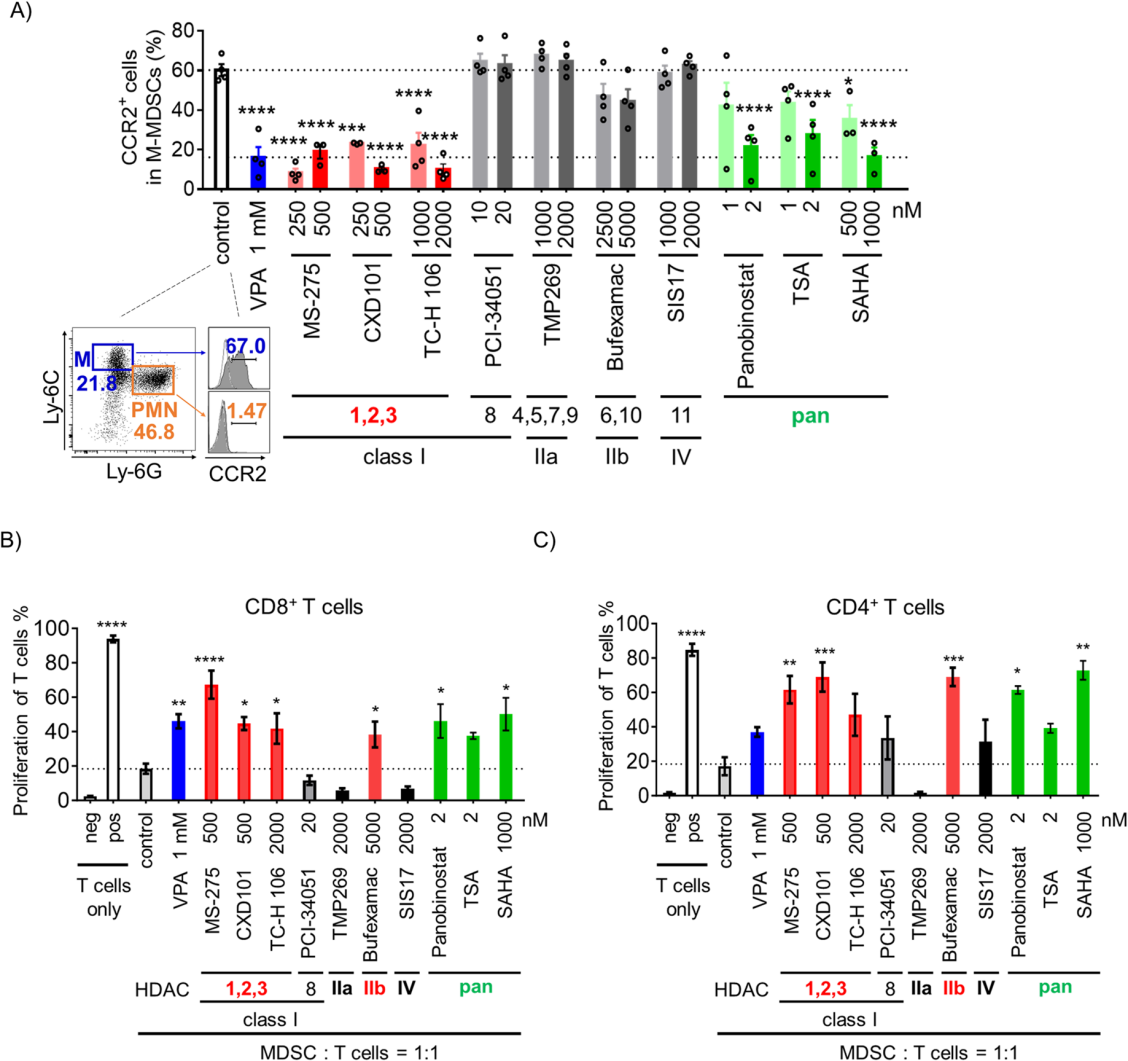
HDAC1-3 inhibitors reduce CCR2 expression and the immunosuppressive function of MDSCs. After 4 days of culture in medium supplemented with GM-CSF (40 ng/mL) with or without the addition of different concentrations of HDAC inhibitors, CCR2 expression on M-MDSCs was assessed using flow cytometry. **A** Gating strategy used for analysis of CCR2 on M-MDSCs and PMN-MDSCs. CCR2 expression in M-MDSCs treated with HDAC inhibitors. Data represent mean ± S.E.M., pooled from three or four independent experiments (*p < 0.05, ***p < 0.001, ****p < 0.0001 by one-way ANOVA, compared with control group). **B**, **C** MDSCs treated with different HDAC inhibitors were combined in a 1:1 ratio with eFluor 670-labeled CD4^+^ or CD8.^+^ T cells, followed by stimulation with anti-CD3ε/anti-CD28 antibodies. Data represent mean ± S.E.M. of three independent experiments (one-way ANOVA: *p < 0.05, **p < 0.01, ***p < 0.001, and ****p < 0.0001, compared with control group). Neg, negative control (T cell only, without anti-CD3ε/anti-CD28 antibodies stimulation); pos, positive control (T cell only, with anti-CD3ε/anti-CD28 antibodies stimulation)

The expression of CCR2, a chemokine receptor on the surface of M-MDSCs, regulates their migration toward tumors and promotes tumor progression [25]. We have previously reported that VPA can significantly reduce CCR2-expressing M-MDSCs, specifically by reducing their accumulation in tumors and inhibiting the growth of lymphoma and melanoma. Therefore, we conducted further studies to investigate HDAC inhibitors that are important for CCR2 expression on MDSCs. Consistent with previous studies, CCR2 was absent in PMN-MDSCs but was present in M-MDSCs (Fig. 1A). VPA treatment significantly reduced the proportion of CCR2^+^ M-MDSCs. HDAC1-3 inhibitors (MS-275, CXD101, and TC-H 106) and pan-HDAC inhibitors reduced the CCR2^+^ population in M-MDSCs, whereas the other HDAC inhibitors exhibited no effects (Fig. 1A). Our results indicate that MS-275 and CXD101 at concentrations below 500 nM, and TC-H 106 at 2000 nM, effectively downregulated CCR2 expression in a dose-dependent manner without significantly affecting cell proliferation (Fig. S1A, B). Thus, HDAC1-3 enhanced CCR2 expression on MDSCs.

We examined the HDAC inhibitors that are important for regulating the immunosuppressive function of MDSCs. As previously reported, control MDSCs without HDAC inhibitors potently inhibited T cell proliferation, whereas pan-HDAC inhibitors, including VPA, reduced the immunosuppressive function of MDSCs (Fig. 1B, C). MDSCs that were stimulated with HDAC1-3 inhibitors but not with HDAC8 inhibitors enhanced T cell proliferation compared to control MDSCs, as did VPA (Fig. 1B, C). MDSCs stimulated with the class IIb inhibitor bufexamac also exhibited increased T cell proliferation compared to control MDSCs. Conversely, MDSCs treated with the class IIa HDAC inhibitor (TMP269-treated MDSCs) suppressed T cell proliferation more than control MDSCs (Fig. 1B, C), indicating that the immunosuppressive function of MDSCs may be increased by class IIa HDAC inhibition. SIS17-treated MDSCs (a class IV HDAC inhibitor) exhibited a stronger suppression of CD8^+^ T cell proliferation than control MDSCs (Fig. 1B). Thus, different HDACs have different effects on the immunosuppressive functions of MDSCs, particularly targeting HDAC1-3 or class IIb HDACs can inhibit MDSC-suppressive activity. Therefore, HDAC1-3 inhibitors decreased CCR2 expression on MDSCs, attenuated their immunosuppressive function, and were considered effective MDSC inhibitors.

### Antitumor effects of HDAC1-3 inhibitors in Hepa 1–6 HCC mouse model

To investigate the potential of HDAC1-3 inhibitions in cancer therapy, we analyzed the expression and prognostic value of HDAC1, 2, and 3 in various cancers using the GEPIA2 web tool based on The Cancer Genome Atlas database [26]. We found that only in liver HCC (LIHC), HDAC1, 2, and 3 expression was significantly associated with survival rate and was higher in tumor tissue than normal tissue (Fig. S2A-G). Using TIMER 2.0 [27], we observed a positive correlation between HDAC1, 2, and 3 expression and tumor-infiltrating MDSCs in LIHC (Fig. S2H). These findings suggest that elevated levels of HDAC1, HDAC2, and HDAC3, possibly linked to MDSC infiltration, may contribute to worsened disease outcomes in LIHC.

**Fig. 2:**
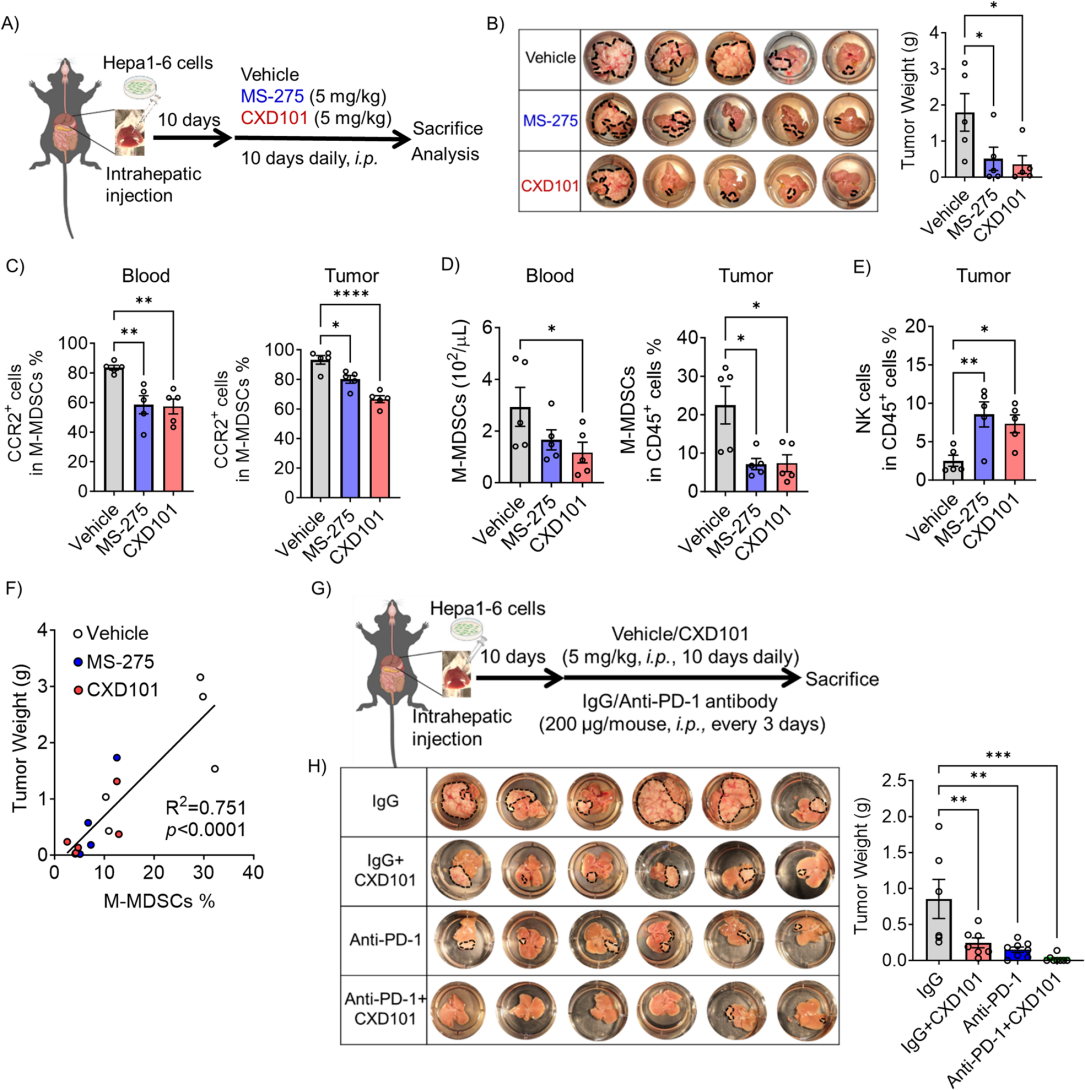
HDAC1-3 inhibitors impaired Hepa 1–6 tumor development. **A** orthotropic mouse model using Hepa 1–6 cells was established and administered drugs as per the dosing schedule. **B** Endpoint tumor weight was measured with representative images displaying tumor morphology. **C** The proportion of CCR2^+^ cells among total M-MDSCs from blood and tumors were assessed using flow cytometry. **D** Flow cytometry of the M-MDSCs (CD11b^+^Ly-6G^−^Ly-6C^hi^) in blood and tumors. **E** Flow cytometry of the tumor NK cells (CD3ε^−^NK1.1^+^) in CD45^+^ live cells. **F** Correlation between M-MDSCs and tumor weight in Hepa 1–6 tumor sites was determined using Pearson’s correlation coefficient test. The above data represent mean ± S.E.M. from one of the representative experiments, with *n* = 5 per group (*p < 0.05, **p < 0.01, ****p < 0.0001 by one-way ANOVA). **G** Ten days after inoculation with Hepa 1–6 cells, the mice were administered intraperitoneal injection of vehicle, MS-275, or CXD101 (5 mg/kg) daily in combination with an anti-PD-1 antibody or IgG (200 µg/mouse) every 3 days until day 20. **H** Endpoint tumor weight was measured with representative images displaying tumor morphology. Data represent mean ± S.E.M. with *n* = 6–8 per group (**p < 0.01, ***p < 0.001 by two-way ANOVA)

To further investigate the antitumor effects of the HDAC1-3 inhibitors, MS-275 and CXD101, Hepa 1–6 orthotropic HCC model mice were used (Fig. 2A). A dosage of 5 mg/kg MS-275 and CXD101 was intraperitoneally administered daily for 10 days to Hepa 1–6 transplanted mice because this dosage significantly reduced M-MDSC CCR2 expression without causing noticeable weight loss in mice (Fig. S3B, Fig. S4E). They were found to inhibit tumor growth compared to that in the control mice (Fig. 2B). These inhibitors decreased CCR2 expression on M-MDSCs from the spleen, blood, and tumors (Fig. 2C, S4A). MS-275 tended to reduce M-MDSCs in the blood and tumors, and CXD101 treatment significantly reduced these numbers (Fig. 2D). However, PMN-MDSCs from each tissue did not exhibit any differences (Fig. S4B). HDAC1-3 inhibitors did not increase CD8^+^ T cells in tumors but significantly increased NK cells (Fig. 2E, S4C). Furthermore, the number of M-MDSCs was positively correlated with tumor weight and negatively correlated with NK cell number, indicating that M-MDSCs are key cells in HCC progression (Fig. 2F, S4D). An independent repeat of the experiment revealed similar trends in the results (Fig. S5). In vitro treatment of Hepa 1–6 cells with MS-275 or CXD101 resulted in inhibition of their proliferation in a dose-dependent manner (Fig. S6). However, at concentrations below 500 nM, they had little effect on cancer cells (Fig. S6) and strongly affected MDSCs (Fig. S1B). Therefore, the anticancer effects of MS-275 and CXD101 in vivo may be mediated by MDSCs rather than by cancer cells. Thus, HDAC1-3 inhibitors may reduce the number of M-MDSCs and relieve the tumor immune suppression microenvironment, thereby limiting HCC progression.

The potential of HDAC1-3 inhibitors to enhance the antitumor effects of anti-PD-1 antibodies was examined. While treatment with CXD101 alone or anti-PD-1 antibody alone significantly inhibited tumor growth, residual tumors persisted. Notably, the combined treatment resulted in 75% (6/8) of tumor-free cases, demonstrating enhanced efficacy (Fig. 2G, H). Therefore, HDAC1-3 inhibitors potentiate the antitumor effects of anti-PD-1 antibodies in an orthotropic HCC mouse model. Additionally, we did not observe any significant weight loss in the mice throughout the treatment period, suggesting that the administered drugs did not have apparent toxic side effects (Fig. S4E).

### ATAC-seq and RNA-seq analyses of M-MDSCs treated with HDAC1-3 inhibitors

**Fig. 3:**
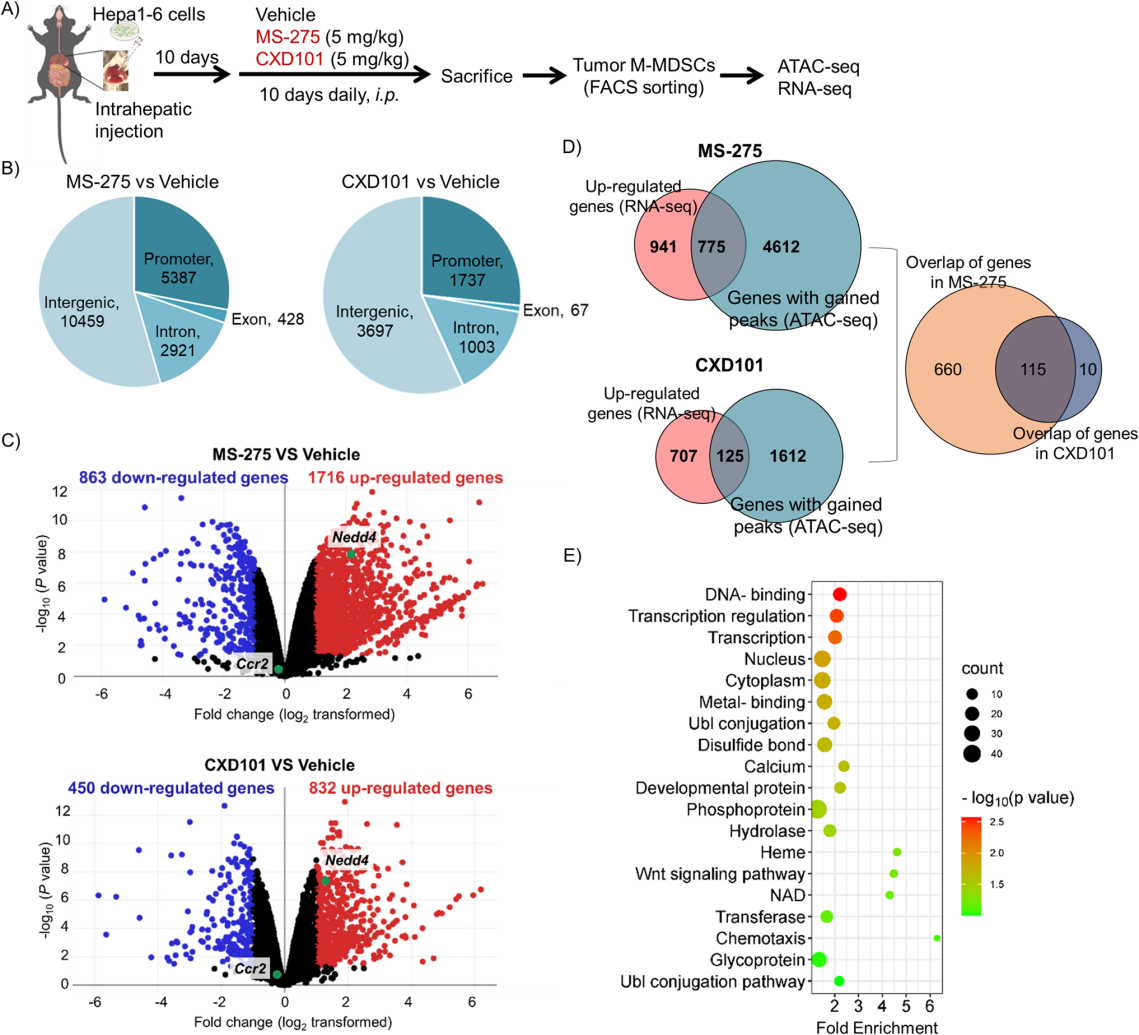
Identifying HDAC1-3 inhibitors-induced M-MDSC epigenetic changes. **A** The drug dosing schedule is shown. M-MDSCs sorted from Hepa 1–6 tumor site were used for ATAC-seq and RNA-seq. **B** Proportions and number of the ATAC-seq peaks annotated to different genomic regions in MS-275 and CXD101 groups versus the vehicle group (*n* = 2 biological replicates). **C** Volcano plots showing differences in mRNA expression profiles of MDSCs (*n* = 2 biological replicates). **D** Venn diagram showing an overlap of genes with significantly gained ATAC-seq signal and upregulated genes (RNA-seq) compared with the vehicle group. **E** DAVID analyses of the identified 115 genes using the UP_KEYWORDS Functional Category (count > 2 with *p* < 0.05)

To further elucidate the molecular mechanisms underlying HDAC1-3 inhibitors by MDSCs, we performed ATAC-seq and RNA-seq analyses of M-MDSCs isolated from Hepa 1–6 tumor-bearing mice treated with MS-275 or CXD101 (Fig. 3A). ATAC-seq identifies open chromatin regions using fragmentation and sequencing. In the MS-275-treated group, there were 19,195 peaks, of which 5387 (28.1%) were in the promoter region (Fig. 3B). In the CXD101-treated group, there were 6437 peaks, and 1737 (26.9%) were in the promoter region (Fig. 3B). RNA-seq was conducted to investigate changes in HDAC expression following MS-275 or CXD101 treatment and to identify genes with open chromatin promoters and increased expression. MS-275 treatment resulted in 1716 upregulated genes compared to those in the vehicle group (Fig. 3C). Intersection with the ATAC-seq analysis yielded 775 common genes (Fig. 3D). CXD101 treatment resulted in the upregulation of 832 genes (Fig. 3C), with ATAC-seq peaks observed in the promoter regions of 125 genes (Fig. 3D). Among these, 115 genes were common to both the MS-275- and CXD101-treated groups and were considered candidates for epigenetic regulation by HDAC1-3 in M-MDSCs (Fig. 3D). Gene ontology enrichment analysis [28] of the 115 identified genes revealed annotations related to transcriptional regulation and ubiquitination (Fig. 3E).

### NEDD4 induces degradative ubiquitination of CCR2

**Fig. 4:**
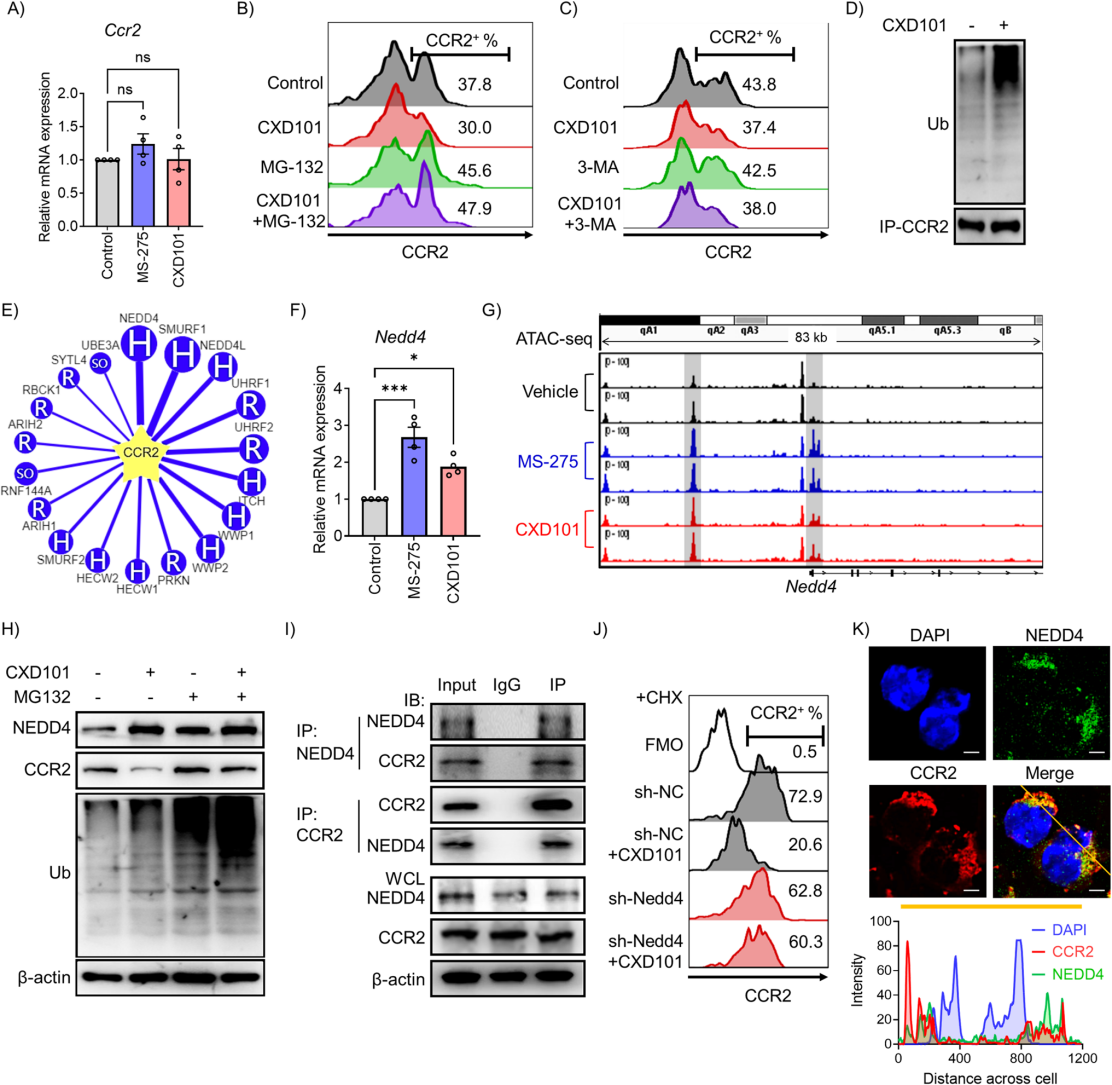
Proteasome-dependent degradation regulates CCR2 expression in M-MDSCs. **A** Total RNA was extracted from in vitro M-MDSCs, and qRT-PCR was performed to measure the mRNA expression of *Ccr2* (*n* = 4 biological replicates). **B** Flow cytometry of CCR2 expression on in vitro M-MDSCs after 6 h of treatment with CXD101 (250 nM), MG-132 (5 µM), or CXD101 and MG-132. **C** Flow cytometry of CCR2 expression on in vitro M-MDSCs after 6 h of treatment with CXD101 (250 nM), 3-MA (1 mM), or CXD101 and 3-MA. **D** In vitro MDSCs treatment with CXD101 (250 nM) for 6 h. Cell lysates were immunoprecipitated with anti-CCR2 and analyzed by immunoblotting with Ubiquitin antibody. **E** The network view of the predicted E3 ligase for CCR2 by UbiBrowser2.0 web server. CCR2 locates in the center of the canvas. The predicted E3 ligases surround the substrate, the edge width and surrounding node size are positively correlated with the confidence of prediction. The letters in node represent different E3 ligase types (H, HETC; R, RING; SO, SINGLE Other). **F** Total RNA was extracted from in vitro M-MDSCs, and qRT-PCR analysis was performed to measure the mRNA expression levels of *Nedd4* (*n* = 4 biological replicates). **G** Normalized ATAC-seq tracks of *Nedd4* locus in Vehicle, MS-275, and CXD101 group. **H** In vitro MDSCs treated with CXD101 (250 nM), MG-132 (5 µM), or CXD101 and MG-132 for 6 h. The protein levels of NEDD4, CCR2, and ubiquitin were analyzed by western blot. **I** Coimmunoprecipitation was conducted with NEDD4 and CCR2 antibodies. The coimmunoprecipitated mixture was separated by SDS-PAGE and evaluated by immunoblotting. **J** Flow cytometry of CCR2 expression on in vitro M-MDSCs after 12 h of treatment with CXD101 (250 nM), sh-NC, or sh-NEDD4. **K** Immunofluorescence assay demonstrated the colocalization of NEDD4 and CCR2 in MDSCs (green NEDD4, red CCR2, and blue DAPI), white scale bars: 10 µm. FMO, Fluorescence Minus One; IP, Immunoprecipitation; IB, immunoblot; WCL, whole cells lysate

We first quantified *Ccr2* mRNA expression in M-MDSCs and found that HDAC1-3 inhibitors did not significantly alter *Ccr2* mRNA expression (Fig. 4A), suggesting that HDAC1-3 inhibitors did not affect *Ccr2* transcriptional activity. We used the proteasome inhibitor MG-132 and revealed that MG-132 prevented the CXD101-induced decrease in CCR2 expression in M-MDSCs (Fig. 4B, H). However, the autophagy inhibitor 3-MA did not prevent the HDAC1-3 inhibitor-induced decrease in CCR2 expression (Fig. 4C). Furthermore, CXD101 treatment increased the ubiquitin level of CCR2 protein (Fig. 4D). These results revealed that HDAC1-3 reduced CCR2 protein levels by degrading it via the ubiquitin–proteasome system.

Ubiquitination is a reversible process in which the removal of ubiquitin chains is facilitated by approximately 90 deubiquitinases (DUBs) in humans. The regulation of protein ubiquitination is achieved through a balance between E3 ligase-mediated ubiquitination and DUB-mediated de-ubiquitination. Of the 115 genes identified in this study, no DUBs were identified, and three E3 ligases were found: *Nedd4*, *Trim6*, and *Trim32*. We used UbiBrowser 2.0 to predict CCR2 E3 ligases and found 18 E3 ligase candidates, among which NEDD4 had the highest confidence score (0.882), while TRIM6 and TRIM32 were not detected (Fig. 4E). *Nedd4* mRNA expression was increased in MS-275- and CXD101-treated M-MDSCs (Fig. 4F) and NEDD4 protein expression was also increased (Fig. 4H). ATAC-seq analysis revealed significant ATAC-seq peaks from 34 bp upstream to 1062 bp downstream of the *Nedd4* transcription start site and around 2200 bp upstream of the transcription start site in MS-275- and CXD101-treated groups, while most controls did not have them (Fig. 4G). The regions with ATAC-seq peaks were also ENCODE Candidate Cis-Regulatory Elements (cCREs) containing promoter and enhancer regions (E0912166/prom, E0912167/enhP, E0912168/enhP, E0912139-E0912141/enhD). Therefore, we further conducted reciprocal Co-IP/western blot assays to confirm again that endogenous NEDD4 and CCR2 interact with each other in the MDSCs (Fig. 4I). Pulse-chase assays with CHX were then conducted to assess the protein stability of CCR2 in the presence and absence of NEDD4. Under CHX treatment, CCR2 levels progressively declined, whereas CXD101 treatment significantly hastened CCR2 degradation (Fig. S7A). Notably, NEDD4 knockdown counteracted the CXD101-induced reduction in CCR2, suggesting a critical role for NEDD4 in CCR2 ubiquitination and subsequent degradation (Fig. S7B-C, 4J). Moreover, immunofluorescence assays showed NEDD4 and CCR2 colocalization in the MDSCs (Fig. 4K). These results indicate that HDAC1-3 inhibitors open chromatin at the *Nedd4* locus and activate its transcription, indicating HDAC1-3 inhibitors derepress *Nedd4* expression and enhance CCR2 ubiquitination and degradation.

## Discussion

MDSCs have been implicated in cancer progression and conferring resistance to chemotherapy, radiotherapy, and ICB therapy. Therefore, the targeting of MDSCs is a significant challenge in cancer immunotherapy. In this study, we examined the epigenetic control mechanisms underlying cell fate and function, and elucidated the role of HDACs in regulating MDSCs. We found that HDAC1-3 inhibition leads to the degradation of CCR2 in M-MDSCs via the ubiquitin–proteasome system. This suggests that the HDAC1-3 inhibitors, MS-275 and CXD101, downregulate CCR2 expression in M-MDSCs and decrease M-MDSCs in tumors. Consistent with our results, MS-275 administration to patients with breast cancer resulted in a greater reduction in M-MDSCs than in PMN-MDSCs [29]. Similar to other cancers [30, 31], VPA or HDAC1-3 inhibitors alone exhibited antitumor effects, which were further enhanced when combined with ICB therapy in HCC. Thus, targeting MDSCs and inhibiting HDAC1-3 are promising strategies for cancer immunotherapy.

CCR2 plays a crucial role in regulating M-MDSC function in cancer by affecting tumor growth, metastasis, and immunotherapy resistance [32–34]. Despite its importance, the molecular mechanisms underlying CCR2 expression remain poorly understood. Study has revealed that FOXO1 directly binds to the CCR2 promoter and activates its transcription [35]. Our findings reveal a novel regulatory mechanism by which the HDAC inhibitors MS-275 and CXD101 do not affect *Ccr2* transcription but instead reduce CCR2 protein levels by enhancing its ubiquitination-mediated degradation. Our results provide novel insights into the intricacies of CCR2 regulation and suggest that HDAC inhibitors hold therapeutic promise for M-MDSC targeting in cancer. In order to identify the E3 ligase responsible for CCR2 ubiquitination, we conducted ATAC-seq and RNA-seq analyses and identified NEDD4 as a candidate gene. NEDD4 is a HECT-type E3 ligase composed of an N-terminal C2 domain, multiple WW domains, and a C-terminal HECT domain. It has been shown to ubiquitinate membrane proteins, including ion channels and transmembrane receptors [36]. Our research found that the WW domain of NEDD4 recognized and interacted with the PPxY motif of CCR2, which is a common target for WW domain-mediated ubiquitination. Additionally, UbiBrowser2.0 predicted that E3 ligases with WW motifs, including all nine members of the NEDD4 family, were likely to ubiquitinate CCR2. To our knowledge, our results mark the initial discovery of the interaction between NEDD4 and CCR2, with NEDD4 serving as a potent E3 ligase for CCR2 ubiquitination.

Our results demonstrated that the inhibition of HDAC1-3 or class IIb HDACs attenuated the immunosuppressive function of MDSCs. Considering VPA lacks class IIb HDAC inhibitory activity, we hypothesized that HDAC1-3 inhibition would reduce MDSC immunosuppression. Additionally, HDAC6 is highly expressed in M-MDSCs and selective inhibitors decrease their immunosuppressive function in tumor-bearing mice [16]. This is consistent with our finding that class IIb HDAC inhibitors, including HDAC6, reduce MDSC immunosuppression. However, the mechanisms through which HDACs regulate the immunosuppressive function of MDSCs remain unclear and require further investigation. Additionally, the differential effects of HDACs on MDSC subtypes and their underlying mechanisms should be explored. In conclusion, our evidence suggests that HDAC1-3 inhibitors decrease CCR2 expression in MDSCs, attenuate their immunosuppressive function, and are effective MDSC inhibitors. Although we could not determine which HDAC1-3 phenotype predominantly influences CCR2 regulation and immunosuppressive function in this study, we aim to generate a conditional knockout mouse model of HDAC in bone marrow cells to clarify its in vivo function for future studies.

While considering the therapeutic effects of HDAC inhibitors, it is imperative to contemplate the potential for off-target effects and their implications on our observed outcomes. To address this concern, we utilized three HDAC1-3 inhibitors and noted consistent modulation of M-MDSCs, suggesting that the observed effects are specific to HDAC1-3 inhibition rather than a broad off-target consequence. Moreover, our inhibitors showed high selectivity within the concentrations used in our experiments (Supplemental Table 1). Acknowledging the necessity for further validation, we propose that future studies could benefit from employing gene editing technologies to directly manipulate HDAC expression. This would allow for an investigation of their effects on M-MDSCs, independent of potential off-target influences, providing a more direct assessment of HDACs’ roles and aiding in the elucidation of the precise mechanisms by which they impact M-MDSCs.

Given the insidious onset of liver cancer, less than 30% of patients are eligible for radical treatment at the time of initial diagnosis, underscoring the crucial role of systemic antitumor therapies in the management of intermediate to advanced stages of the disease. Current systemic antitumor approaches encompass immune checkpoint inhibitors, anti-VEGF therapies, and tyrosine kinase inhibitors [17]. Multiple clinical studies have demonstrated that combination therapies with these agents yield superior long-term outcomes compared to traditional monotherapy with the sorafenib, thereby establishing them as the preferred first-line treatment options [37]. Our study introduces HDAC1-3 inhibitors, such as CXD101, as a novel therapeutic alternative. We discovered that the combination of HDAC1-3 inhibitors with anti-PD-1 antibodies induced an almost complete regression of orthotropic Hepa 1–6 tumors in mice. This antitumor effect could be facilitated by the HDAC1-3-mediated reduction of MDSC and the enhancement of NK cell activation. The rationale for combining CXD101 with anti-PD-1 therapies is grounded in evidence that MDSCs contribute to resistance to anti-PD-1 treatments by suppressing T cell activation. By reducing M-MDSC infiltration, CXD101 can counteract this suppression, potentially enhancing the efficacy of anti-PD-1 antibodies. This synergistic approach targets both the immunosuppressive cells within tumor microenvironment and the immune checkpoint pathway, addressing a common resistance mechanism to immune checkpoint inhibitors. These discoveries not only reveal a novel mechanism underlying the action of HDAC inhibitors in HCC but also suggest a promising combination strategy employing anti-PD-1 antibodies for clinical translation.

Notably, our findings are based on a single tumor model, which may not capture the full spectrum of biological responses observed across various species and models. This limitation underscores the importance of validating our results in additional animal models to enhance the generalizability of our conclusions. Furthermore, translating our preclinical findings to clinical applications necessitates validation in human samples. Future studies will focus on investigating the impact of HDAC inhibitors on human MDSCs, which is crucial for establishing the clinical relevance of our research.

## Supplementary Information

Below is the link to the electronic supplementary material.

Supplementary file1 (PDF 879 KB)

## Data Availability

NGS data are available in GEO under the accession number GSE232268. All data are available from the corresponding authors upon request.
