# Supplementary material for: HDAC1-3 inhibition triggers NEDD4-mediated CCR2 downregulation and attenuates immunosuppression in myeloid-derived suppressor cells
Source: Cancer Immunol Immunother. 2025 Feb 1;74(3):81. doi: 10.1007/s00262-024-03931-y (PMC11787094; doi:10.1007/s00262-024-03931-y)
Supplement: Supplementary file 1 — Supplementary file1 (PDF 879 KB) [file 262_2024_3931_MOESM1_ESM.pdf]

Supplemental Data

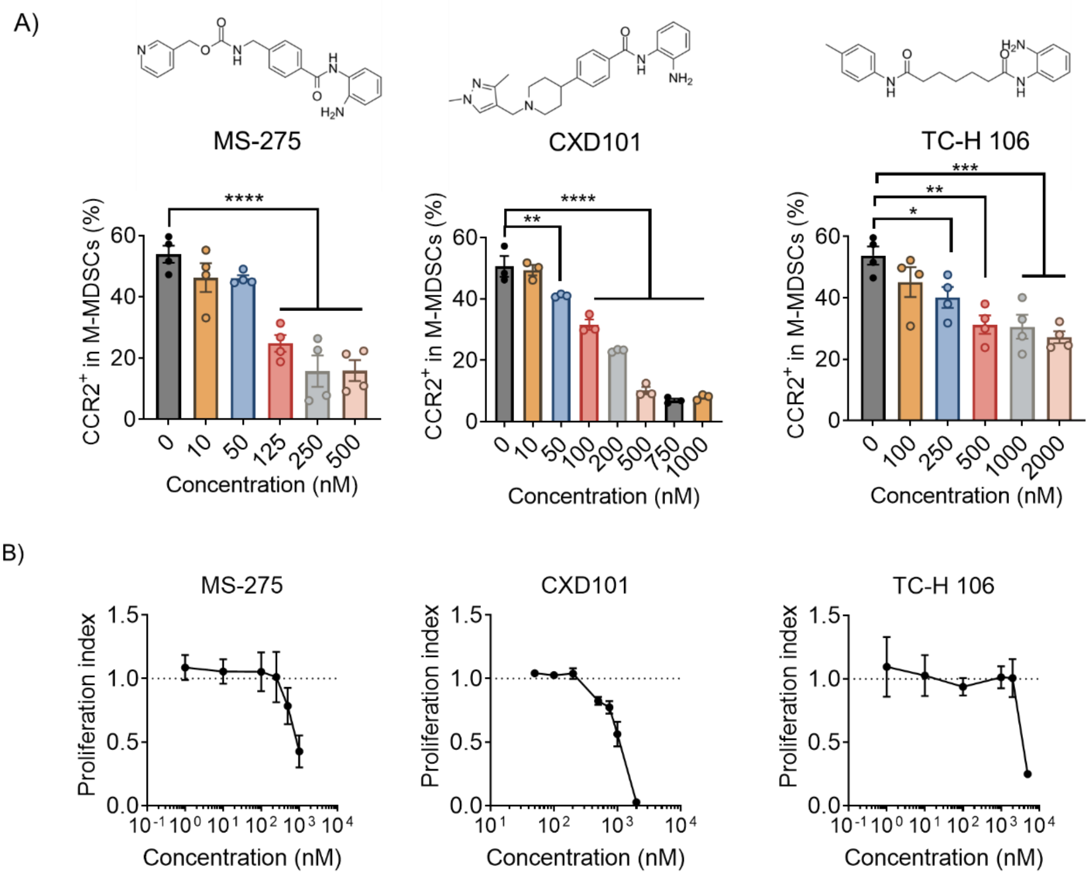

Fig. S1 The proliferation of MDSCs following with HDAC inhibitor treatment. BM cells cultured in media supplemented with GM-CSF (40 ng/mL) with or without the addition of different concentrations of HDAC inhibitors for 4 days. **A)** Molecular structures of HDAC1, 2, 3 inhibitors (MS-275, CXD101, and TC-H 106) are shown above. CCR2 expression on M-MDSCs treated with different concentrations of the HDAC inhibitors was assessed. Data represent mean ± S.E.M., pooled from two independent experiments (**p* < 0.05, ***p* < 0.01, ****p* < 0.001, *****p* < 0.0001 by one-way ANOVA, compared with the group without additional HDAC inhibitor). **B)** The number of MDSCs was determined using flow cytometry with precision count beads. The proliferation index was calculated as a ratio of the number of MDSCs treated with HDAC inhibitor to that in non-treated group. Data represent mean ± S.E.M., pooled from three independent experiments.

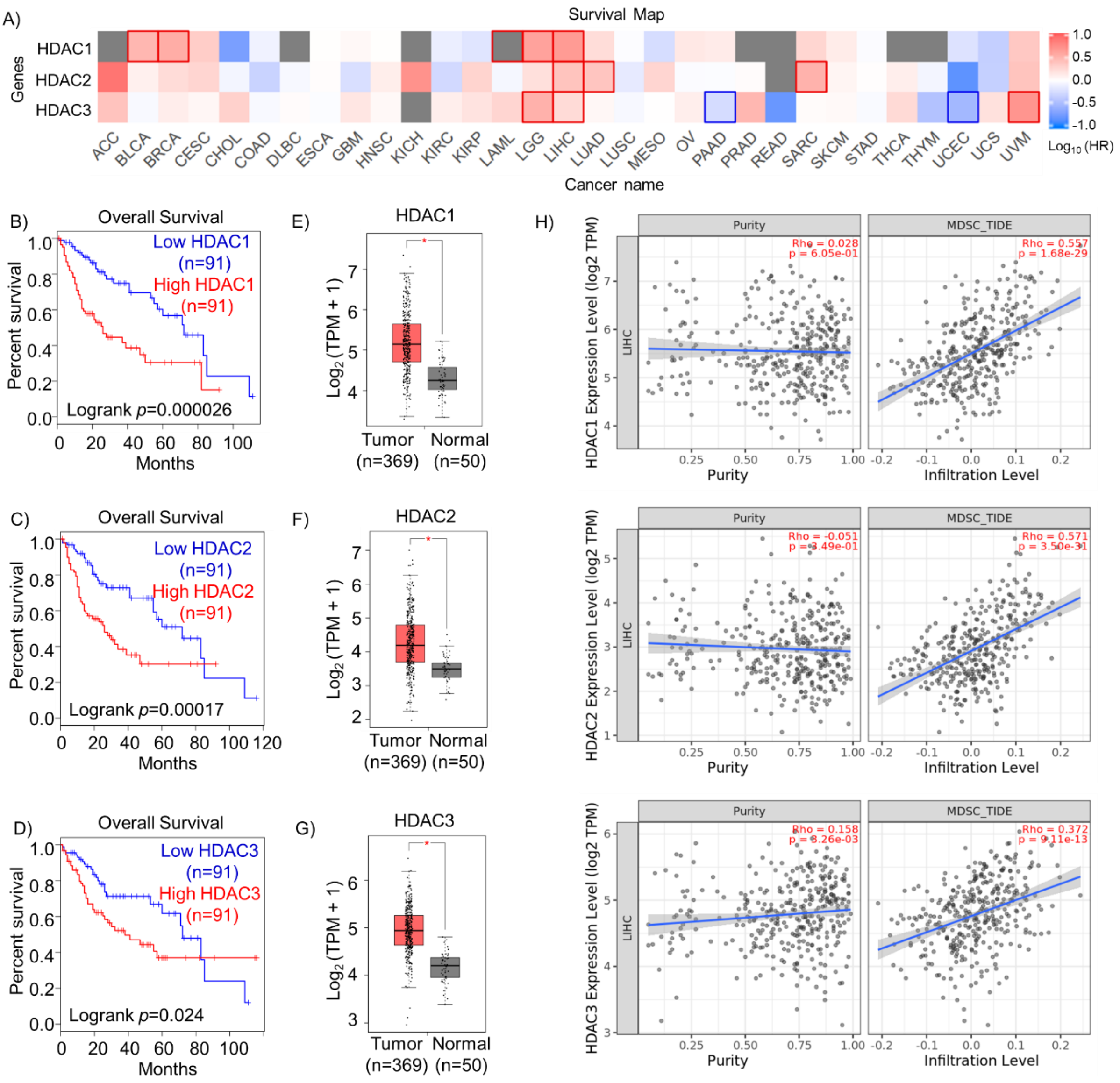

Fig. S2 High expression HDAC1, 2, 3 are related with poor prognosis of HCC. A) The survival contribution of *Hdac1*, *Hdac2*, and *Hdac3* in multiple cancer types was assessed by the GEPIA2 web server. The survival heat map shows the hazard ratios (HR) in logarithmic scale (log10) for different genes. The red and blue blocks indicate higher and lower risks, respectively. The rectangles with frames indicate the significant results in prognostic analyses (quartile cut-off, $p < 0.05$ using the Mantel–Cox test). **B-D)** The overall survival curves comparing the high (red line) and low (blue line) (quartile cut-off) expressions of *Hdac1*, *Hdac2*, and *Hdac3* in liver hepatocellular carcinoma (LIHC) in the GEPIA2 web server. **E-G)** The expression of *Hdac1*, *Hdac2*, and *Hdac3* in the LIHC tissue group compared with the normal tissue group. Asterisk represents fold change > 1.5 with $p < 0.01$. The dots represent expression in each sample. **H)** Correlation of *Hdac1*, *Hdac2*, and *Hdac3* expression with tumor purity (the proportion of cancer cells in a sample; left) and with the infiltration level of MDSCs (right) analyzed by the TIMER2.0 web server in LIHC.

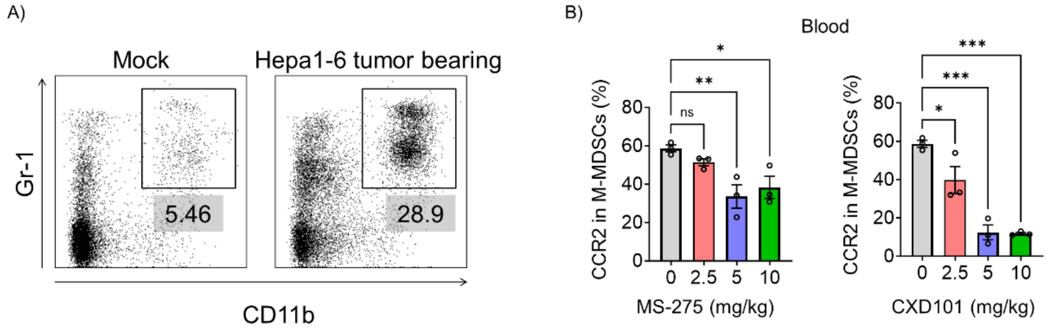

Fig. S3 A) The proportion of $CD11b^+Gr-1^+$ cells in the blood was analyzed after 10 days of Hepa 1-6 inoculation. **B)** Different dosage of MS-275 and CXD101 were administered in the Hepa 1-6 tumor bearing mice on day10. The proportion of $CCR2^+$ cells among M-MDSCs from blood were assessed using flow cytometry.

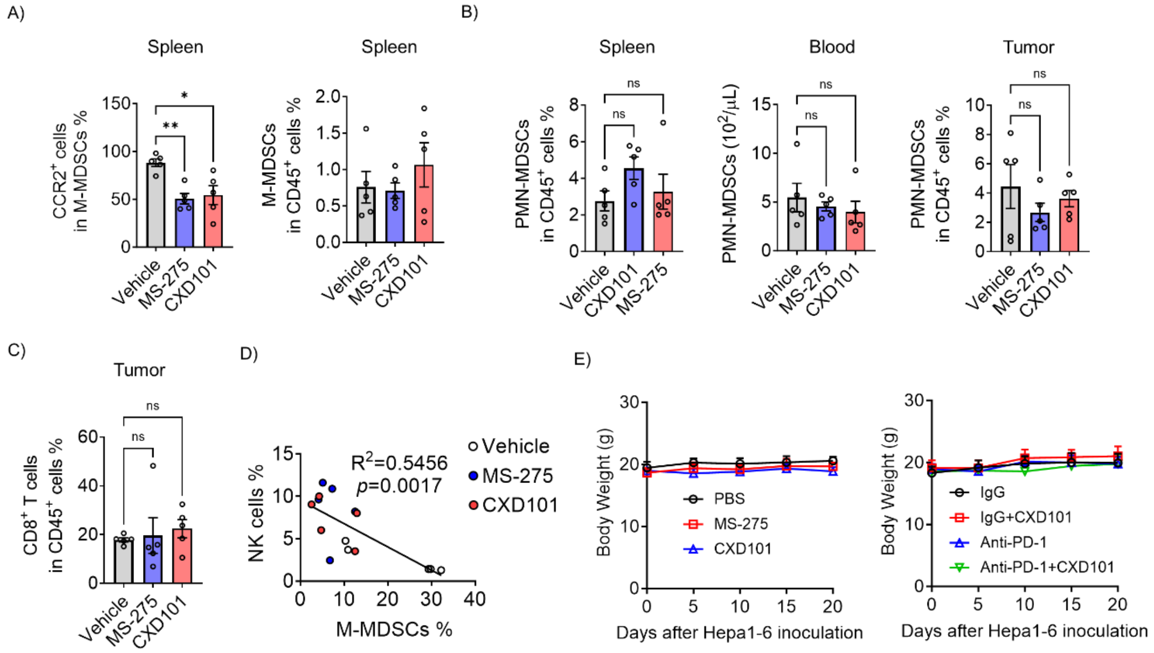

Fig. S4 A) The proportion of $CCR2^+$ cells among total M-MDSCs and the M-MDSCs ($CD11b^+Ly\text{-}6G^-Ly\text{-}6C^{hi}$) from spleen using flow cytometry. **B)** Flow cytometry of the PMN-MDSCs ($CD11b^+Ly\text{-}6G^+Ly\text{-}6C^{int}$) in spleen, blood, and tumors. **C)** Flow cytometry of the proportion of tumor $CD8^+$ T cells ($CD3\varepsilon^+NK1.1^-CD8\alpha^+$) in $CD45^+$ live cells. **D)** Correlation between M-MDSCs and NK cells in Hepa 1-6 tumor sites was determined using Pearson's correlation coefficient test. **E)** Average body weights of mice post Hepa1-6 inoculation. Data represent mean ± S.E.M one representative experiment. (*$p < 0.05$, ** $p < 0.01$ by one-way ANOVA).

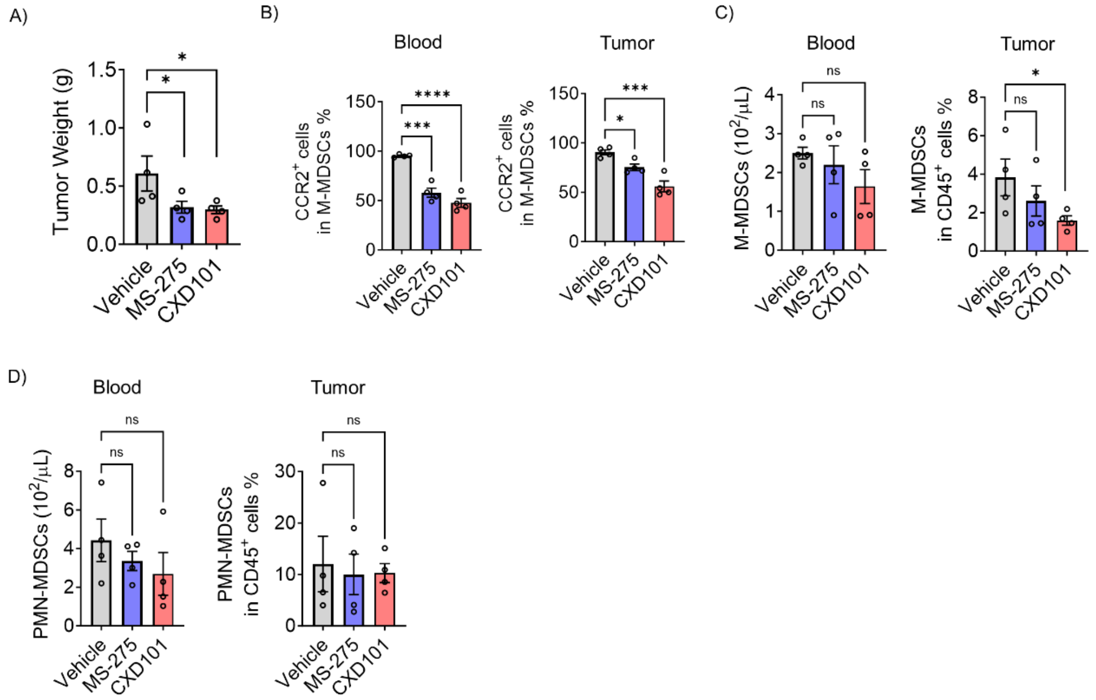

Fig. S5 An independent repeat of the experiment to investigate the antitumor effects of MS-275 and CXD101 in the Hepa 1-6 orthotopic HCC model mice, as shown in Fig. 2A. **A)** Tumor weight was measured. **B)** The proportion of $CCR2^+$ cells among total M-MDSCs from blood and tumors were assessed using flow cytometry. **C)** Flow cytometry of the M-MDSCs ($CD11b^+Ly\text{-}6G^-Ly\text{-}6C^{hi}$) in blood and tumors. D) Flow cytometry of the PMN-MDSCs ($CD11b^+Ly\text{-}6G^+Ly\text{-}6C^{int}$) in blood and tumors. Data represent mean ± S.E.M. Data are presented as mean ± S.E.M. with n = 4 per group (*$p < 0.05$, ***$p < 0.001$, ****$p < 0.0001$ by one-way ANOVA).

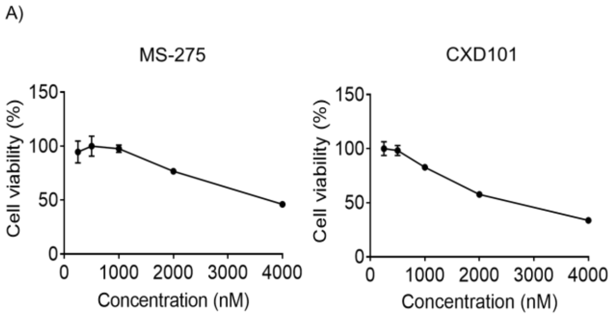

Fig. S6 A) Hepa 1-6 cells treated with MS-275 or CXD101 for 48 h at various concentrations *in vitro* and cell viability was then measured using the CCK-8 kit.

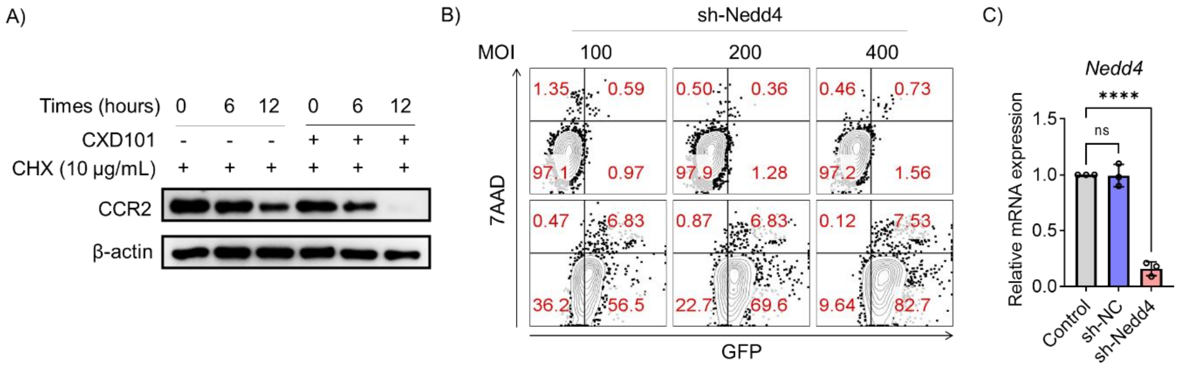

Fig. S7 A) *In vitro* MDSCs treated with CXD101 (250 nM) and CHX (10 μg/mL) for 12 h. The protein levels of CCR2 were analyzed by western blot. **B-C)** BM cells transduced with lentiviruses supplemented with HitransG A and GM-CSF (40 ng/mL) for 72 hours, the efficiency of shRNA was determined by GFP expression and qRT-PCR for *Nedd4* knockdown. ****$p < 0.001$ by one-way ANOVA.

Supplemental table 1. Inhibitory effects of HDACi on HDAC classes.

HDAC / Inhibitor	Class I				IIa				IIb		IV	concentration	reference
	HDAC1	HDAC2	HDAC3	HDAC8	HDAC4	HDAC5	HDAC7	HDAC9	HDAC6	HDAC10	HDAC11		
VPA	700	800	1000	7442	1500	1000	1300	unk.	>200000	>200000	unk.	µM(IC50)	[1]
MS-275	243	453	248	>10000	>10000	>10000	>10000	>10000	>10000	>10000	649	nM(IC50)	[2, 3]
CXD101	63	570	550	unk.	NA	NA	NA	NA	NA	unk.	unk.	nM(IC50)	[4]
TC-H 106	150	760	370	5000	NA	NA	NA	unk.	unk.	unk.	unk.	nM(IC50)	[2]
PCI-34051	4000	>50000	>50000	10	unk.	unk.	unk.	unk.	2900	13000	unk.	nM(IC50)	[5]
TMP269	>100000	>100000	>100000	4200	157	97	43	23	8200	>100000	>100000	nM(IC50)	[6]
Bufexamac	unk.	unk.	341000	235000	unk.	unk.	unk.	unk.	10700	12300	unk.	nM(Kd^{app})	[7]
SIS17	>100000	>100000	>100000	>100000	>100000	>100000	>100000	>100000	>100000	>100000	830	nM(IC50)	[8, 9]
Panobinostat	2.5	13.2	2.1	277	203	7.8	531	5.7	10.5	2.3	2.7	nM(IC50)	[10]
TSA	5	14	9	180	6590	2170	3630	7140	1	14	13	nM(IC50)	[6]
SAHA	38	144	6	38	>30000	>30000	>30000	>30000	10	21	28	nM(IC50)	[3]

NA: not available, represents that the detection range has been exceeded, and it can be considered as having no inhibitory effect. Unk.: unknown.

Reference:

[1] Gurvich, Nadia et al. "Histone deacetylase is a target of valproic acid-mediated cellular differentiation." *Cancer research* vol. 64,3 (2004): 1079-86. doi:10.1158/0008-5472.can-03-0799

[2] Lauffer, Benjamin E L et al. "Histone deacetylase (HDAC) inhibitor kinetic rate constants correlate with cellular histone acetylation but not transcription and cell viability." *The Journal of biological chemistry* vol. 288,37 (2013): 26926-43. doi:10.1074/jbc.M113.490706

[3] Ning, Zhi-Qiang et al. "Chidamide (CS055/HBI-8000): a new histone deacetylase inhibitor of the benzamide class with antitumor activity and the ability to enhance immune cell-mediated tumor cell cytotoxicity." *Cancer chemotherapy and pharmacology* vol. 69,4 (2012): 901-9. doi:10.1007/s00280-011-1766-x

[4] Eyre, Toby A et al. "A phase 1 study to assess the safety, tolerability, and pharmacokinetics of CXD101 in patients with advanced cancer." *Cancer* vol. 125,1 (2019): 99-108. doi:10.1002/cncr.31791

[5] Balasubramanian, S et al. "A novel histone deacetylase 8 (HDAC8)-specific inhibitor PCI-34051 induces apoptosis in T-cell lymphomas." *Leukemia* vol. 22,5 (2008): 1026-34. doi:10.1038/leu.2008.9

[6] Lobera, Mercedes et al. "Selective class IIa histone deacetylase inhibition via a nonchelating zinc-binding group." *Nature chemical biology* vol. 9,5 (2013): 319-25. doi:10.1038/nchembio.1223

[7] Bantscheff, Marcus et al. "Chemoproteomics profiling of HDAC inhibitors reveals selective targeting of HDAC complexes." *Nature biotechnology* vol. 29,3 (2011): 255-65. doi:10.1038/nbt.1759

[8] Son, Se In et al. "Activity-Guided Design of HDAC11-Specific Inhibitors." *ACS chemical biology* vol. 14,7 (2019): 1393-1397. doi:10.1021/acschembio.9b00292

[9] Baselious, Fady et al. "Utilization of an optimized AlphaFold protein model for structure-based design of a selective HDAC11 inhibitor with anti-neuroblastoma activity." *Archiv der Pharmazie* vol. 357,10 (2024): e2400486. doi:10.1002/ardp.202400486

[10] Atadja, Peter. "Development of the pan-DAC inhibitor panobinostat (LBH589): successes and challenges." *Cancer letters* vol. 280,2 (2009): 233-41. doi:10.1016/j.canlet.2009.02.019
